# On the Role of the Ventromedial Prefrontal Cortex in Self-Processing: The Valuation Hypothesis

**DOI:** 10.3389/fnhum.2013.00372

**Published:** 2013-07-10

**Authors:** Arnaud D’Argembeau

**Affiliations:** ^1^Department of Psychology – Cognition and Behavior, University of Liège, Liège, Belgium; ^2^Cyclotron Research Centre, University of Liège, Liège, Belgium

**Keywords:** self, identity, value, significance, medial prefrontal cortex, ventromedial prefrontal cortex, fMRI

## Abstract

With the development of functional neuroimaging, important progress has been made in identifying the brain regions involved in self-related processing. One of the most consistent findings has been that the ventromedial prefrontal cortex (vMPFC) is activated when people contemplate various aspects of themselves and their life, such their traits, experiences, preferences, abilities, and goals. Recent evidence suggests that this region may not support the act of self-reflection *per se*, but its precise function in self-processing remains unclear. In this article, I examine the hypothesis that the vMPFC may contribute to assign personal value or significance to self-related contents: stimuli and mental representations that refer or relate to the self tend to be assigned unique value or significance, and the function of the vMPFC may precisely be to evaluate or represent such significance. Although relatively few studies to date have directly tested this hypothesis, several lines of evidence converge to suggest that vMPFC activity during self-processing depends on the personal significance of self-related contents. First, increasing psychological distance from self-representations leads to decreased activation in the vMPFC. Second, the magnitude of vMPFC activation increases linearly with the personal importance attributed to self-representations. Third, the activity of the vMPFC is modulated by individual differences in the interest placed on self-reflection. Finally, the evidence shows that the vMPFC responds to outer aspects of self that have high personal value, such as possessions and close others. By assigning personal value to self-related contents, the vMPFC may play an important role in the construction, stabilization, and modification of self-representations, and ultimately in guiding our choices and decisions.

## Introduction

As James (1890) pointed out in his insightful piece on the self, each of us inevitably makes a fundamental division of his or her subjective world into two halves, establishing a distinction between what is considered as “me” (or “mine”) and what is considered as “not-me” (or “not-mine”). James further emphasized that the two sides of the division are far from being treated equally: “the altogether unique kind of interest which each human mind feels in those parts of creation which it can call *me* or *mine* may be a moral riddle, but it is a fundamental psychological fact” (p. 289). This idea that we attach unique significance to self-related contents may prove useful for interpreting one of the most consistent findings that has emerged from neuroimaging research on self-processing. Over the past decade, a growing number of studies have shown that the ventromedial prefrontal cortex (vMPFC) is activated when people contemplate various aspects of themselves and their life, such their traits, experiences, preferences, abilities, and goals (Northoff et al., 2006; Lieberman, 2010; D’Argembeau and Salmon, 2012; Wagner et al., 2012; Martinelli et al., 2013). However, while it is now common to see the vMPFC referenced as a “self region,” the precise mental operations mediated by this area remain poorly understood. Currently, there is no consensus on what this region really does when people think about themselves (for different views, see e.g., Amodio and Frith, 2006; Schmitz and Johnson, 2007; Legrand and Ruby, 2009; Mitchell, 2009; Northoff et al., 2011; Lieberman, 2012).

In this article, I examine the hypothesis that the vMPFC may contribute to generating the “unique kind of interest” in self-related contents that William James emphasized. Many studies have shown that the vMPFC plays a key role in representing the affective significance or subjective value of various types of stimuli (for review, see Rangel and Hare, 2010; Levy and Glimcher, 2012; Roy et al., 2012). Most of these studies focused on the processing of stimuli from the external environment that, at first sight, have nothing to do with self-representations. Could it be, however, that the vMPFC plays a similar role in self-processing? In other words, could it be that the vMPFC contributes to assign value or significance to self-related contents? Before examining this hypothesis, I first specify what is meant by “self” in this context and then provide an overview of functional neuroimaging studies showing the involvement of the vMPFC in self-processing.

## The Multifaceted Self

Any attempt at synthesizing the numerous definitions and conceptualizations of the self that have been proposed in various fields – including philosophy, anthropology, sociology, psychology, and psychiatry – can easily become a daunting task. Yet it is important to clarify what one means by “self” in order to avoid any misunderstanding about the implications of neuroimaging findings on this topic (Zahavi and Roepstorff, 2011). Although there is debate on how best to characterize different components of the self, there is some consensus on the idea that the self is not a single entity, but instead a construct that encompasses multiple facets that are supported by distinct processes (Neisser, 1988; Damasio, 1999; Gallagher, 2000; Leary and Tangney, 2003; Morin, 2006; Klein and Gangi, 2010). Within this multi-component framework, one can draw a broad distinction between two main aspects of self: the self as experiencing subject (i.e., the consciousness of oneself as an immediate subject of experience, which generates a sense of personal agency and ownership over behavioral actions and sensory representations) and the self as object of knowledge (i.e., the representation and evaluation of one’s personal characteristics and experiences) (James, 1890; Damasio, 1999; Gallagher, 2000; Legrand, 2007; Klein, 2012; Prebble et al., 2013).

Most psychological and cognitive neuroscience investigations to date have focused on the self as object of knowledge (Legrand and Ruby, 2009; Christoff et al., 2011; Klein, 2012), and this is the aspect of self that is addressed in the current article. The self as object is itself composed of multiple systems or components, including the ability to recognize one’s physical appearance (Devue and Brédart, 2011), representations of one’s personality traits and other personal attributes (Klein and Lax, 2010), memories of one’s past experiences and knowledge of facts about one’s life (Conway, 2005; Renoult et al., 2012), representations of personal goals and projected future experiences (Markus and Nurius, 1986; D’Argembeau et al., 2012b). The self-as-object can also be conceived as including stimuli that are not, strictly speaking, part of the individual but that somehow relate or belong to the self, such as close others and possessions (James, 1890; Belk, 1988; Aron et al., 2004). Although under normal circumstances these different constituents of the self-as-object interact with each other, they are at least partly dissociable (i.e., one component can operate independently from another). For example, there is substantial evidence that knowledge of one’s personality traits is functionally independent from memories of one’s past experiences (for review, see Klein et al., 2008).

## Medial Prefrontal Involvement in Processing Self-Related Contents

The self and its different components are in all likelihood not “located” in a single place in the brain, but may instead depend on distributed neural systems that include both cortical and subcortical structures (Northoff and Panksepp, 2008; Damasio, 2010). Quite remarkably, however, there is growing evidence that the processing of various types of self-related contents – which form parts of the self-as-object – is commonly associated with activation of the medial portion of the prefrontal cortex (for recent reviews and meta-analyses, see Northoff et al., 2006; van der Meer et al., 2010; Qin and Northoff, 2011; Denny et al., 2012; D’Argembeau and Salmon, 2012; Murray et al., 2012; Wagner et al., 2012; Martinelli et al., 2013).

The representation of one’s personality traits is the aspect of self that has been most frequently investigated in functional neuroimaging studies. In a typical study (see e.g., Kelley et al., 2002), the brain activity associated with evaluating the self-descriptiveness of personality traits (e.g., polite, dependable, daring) is compared to the activity associated with making the same kind of judgments in reference to another person. Several dozen studies using this paradigm have been published to date, and two recent meta-analyses have shown that the medial prefrontal cortex (MPFC)^1^ is the brain region that is most consistently activated during trait self-judgments (van der Meer et al., 2010; Murray et al., 2012). Activations in this region have been observed across different age groups, including children (Pfeifer et al., 2007), adolescents (Schneider et al., 2012), and young and older adults (Gutchess et al., 2007; Ruby et al., 2009). The evidence further suggests that the MPFC is involved in representing and evaluating a variety of different types of personal characteristics, not only one’s personality traits but also one’s attitudes, values, mental states, and physical attributes (e.g., Zysset et al., 2002; Jenkins and Mitchell, 2011; Brosch et al., 2012).

The neural basis of autobiographical memory – memories of one’s past experiences and knowledge of facts about one’s life – has also received extensive attention (for review, see Maguire, 2001; Cabeza and St Jacques, 2007; Piolino et al., 2009). In many studies, memories of specific personal experiences (i.e., events that happened at a particular place and time in an individual’s life) are compared with the retrieval of non-personal information (e.g., non-personal semantic knowledge or stimuli that have been learned in the laboratory before the scanning session). Several meta-analyses have shown that the MPFC is one of the brain regions most commonly activated during autobiographical memory retrieval, along with medial and lateral temporal cortices, the posterior cingulate/retrosplenial cortex, and the inferior parietal lobe (Gilboa, 2004; Svoboda et al., 2006; McDermott et al., 2009; Spreng et al., 2009; Kim, 2012; Martinelli et al., 2013). Of particular interest, a recent meta-analysis has further revealed that the MPFC is the only brain region that is consistently activated when thinking about one’s traits, retrieving specific experiences from one’s past, and accessing knowledge of facts about one’s life, with both common and distinct MPFC activations across these three kinds of self-related information (Martinelli et al., 2013).

Besides memories and knowledge of one’s past, an important part of self-representation refers to one’s personal goals and projected future experiences (Markus and Nurius, 1986; Schacter et al., 2008; Szpunar, 2010; Rathbone et al., 2011; D’Argembeau et al., 2012b). In this regard, a number of studies have shown that the MPFC is activated when people think about goal states such as their hopes and aspirations (Johnson et al., 2006, 2009; Mitchell et al., 2009; Packer and Cunningham, 2009). In recent years, there has also been a growing interest in the concept of episodic future thought – the ability to imagine or simulate specific events that might occur in one’s personal future (Schacter et al., 2008; Szpunar, 2010) – and there is now substantial evidence that episodic remembering and future thinking largely depend on the same core network of brain regions, among which the MPFC is a key player (e.g., Addis et al., 2007; Sharot et al., 2007; Szpunar et al., 2007; Botzung et al., 2008; for review, see Schacter et al., 2012). Of interest is the finding that the MPFC is more activated when thinking about one’s personal past and future than when contemplating the non-personal past and future (Abraham et al., 2008). Furthermore, it has been shown that envisioning events in one’s personal future and reflecting on one’s personality traits are associated with overlapping activation in the MPFC (D’Argembeau et al., 2010a), which provides additional evidence that this region is involved in processing different types of self-related information.

A question that has been debated is whether the MPFC is specifically recruited for processing self-related information or whether this region is also involved in processing information about other individuals (Gillihan and Farah, 2005; Legrand and Ruby, 2009; Wagner et al., 2012). There is evidence that self- and other-related judgments are associated with overlapping activation in the MPFC, suggesting that this region may play a broad role in social cognition (see e.g., Van Overwalle, 2009; Denny et al., 2012). Yet, when the two kinds of judgments are directly compared to each other, self-related judgments generally lead to greater activation than other-related judgments, especially in the vMPFC. For example, two recent quantitative meta-analyses have shown that the evaluation of one’s own personality traits is associated with greater vMPFC activation compared to the evaluation of the traits of another person (van der Meer et al., 2010; Murray et al., 2012). In fact, there seems to be a ventral-dorsal gradient in MPFC such that increasingly ventral regions of MPFC are more strongly associated with making judgments about the self, whereas increasingly dorsal regions of MPFC are more strongly involved in making judgments about others (Denny et al., 2012).

A key dimension that influences vMPFC activity when thinking about others is the closeness of the person to oneself; for example, it has been shown that the vMPFC responds more strongly to friends than strangers (Krienen et al., 2010). Studies that have directly compared self-referential judgments with judgments about close others have yielded somewhat inconsistent findings, with some studies observing greater vMPFC activation for self relative to close others (Heatherton et al., 2006; D’Argembeau et al., 2007, 2008; Benoit et al., 2010; Krienen et al., 2010), whereas other studies found comparable levels of activation (Ochsner et al., 2005; Vanderwal et al., 2008). One possible interpretation of these divergent findings is that the differential activation of the vMPFC during self- and other-processing depends on the degree of inclusion of the close other in one’s sense of self. As briefly mentioned above, people’s identities not only include elements that are unambiguously part of them (e.g., their body and mental states) but also outer aspects of their lives, such as their family, friends, and possessions (James, 1890; Belk, 1988). Notably, research has shown that people tend to treat the resources, perspectives, and identities of close others as their own, and that these effects depend on the extent to which the person is included in their sense of self (Aron et al., 2004). Interestingly, it has been found that the strength of activation of the vMPFC when making judgments about the self versus one’s best friend depends on perceived self-other similarity: participants who perceived themselves as more similar to their friend exhibited less differential activation between the two kinds of judgments (Benoit et al., 2010). This finding suggests that the degree of inclusion of close others in the self is an important determinant of the vMPFC response during self- and other-processing (see also Zhu et al., 2007).

Outer aspects of self such as one’s group membership and possessions have also been associated with increased activation in the vMPFC. Morrison et al. (2012) compared the neural activity associated with categorizing in-group and out-group words (i.e., groups participants felt they belonged to vs. groups they felt they did not belong to) to identify the brain regions that responded to one’s group membership. They found that the vMPFC showed increased activity in response to in-group words compared to out-group words (see also Volz et al., 2009, for evidence that more dorsal regions of MPFC also contribute to social identity processes). Kim and Johnson (2012) investigated the brain regions supporting the incorporation of external objects in the self. Participants saw pictures of objects (e.g., clothing, electronic articles) that were either assigned to themselves or to another person. Objects were presented on the screen and participants were cued to place each object either in a basket labeled “mine” or in a basket labeled with the name of another person (“Alex”), and they were asked to imagine owning the objects that were assigned to the self. When contrasting the two kinds of objects, the authors found greater activation in the vMPFC for objects assigned to the self compared to objects assigned to the other person. Furthermore, the vMPFC region that was responsive to self-related objects was also more activated when participants evaluated their own personality traits (compared with the traits of another person) in a separate task. These findings suggest that external objects that have been associated with the self modulate activity in the same vMPFC region as do internal self-representations (see also Kim and Johnson, in press).

In summary, the studies reviewed in this section show that the medial portion of the prefrontal cortex, and especially the vMPFC, is commonly activated when people process a variety of different kinds of self-related information – their traits, attitudes, values, physical attributes, goals, memories, future thoughts, close others, social groups, and possessions. One should note that I focused on the vMPFC because this is the region that has been most consistently associated with elements of the self-as-object, but of course this is not the only brain area involved in processing self-related contents. Other regions that are commonly recruited include the dMPFC, posterior cingulate cortex (PCC), inferior frontal cortex, insula, and regions in medial and lateral temporal cortices (van der Meer et al., 2010; Denny et al., 2012; Murray et al., 2012; Martinelli et al., 2013). The vMPFC is structurally and functionally connected to multiple brain regions (Buckner et al., 2008), and likely interacts with distinct areas and networks depending on the type of self-related information that is processed at a given moment (see e.g., Andrews-Hanna et al., 2010b; Martinelli et al., 2013).

## What is the Role of the vMPFC in Self-Processing?

While there is substantial evidence that the vMPFC is activated when people contemplate self-related contents, the precise role of this region in self-processing is not well understood and remains controversial (see e.g., Legrand and Ruby, 2009). Recent findings suggest that the act of self-reflection in itself may not depend on the vMPFC. Indeed, the vMPFC responds to self-related contents even in the absence of explicit self-referential judgments (Moran et al., 2009; Rameson et al., 2010; Kim and Johnson, in press), and a recent case study has shown that a patient with extensive brain damage to the vMPFC has a largely preserved self-concept and intact introspective and metacognitive abilities (Philippi et al., 2012). Such evidence suggests that while the vMPFC participates in the processing of self-related contents (as shown by the neuroimaging studies reviewed in the previous section), this region may not support the formation of self-representations *per se*. So what might be the function of the vMPFC during self-processing?

### The valuation hypothesis

Activity changes in the vMPFC are not restricted to tasks requiring the processing of self-related contents. Indeed, the vMPFC appears to play a broad role in affective and value-based processing (Phan et al., 2002; Bechara and Damasio, 2005; Kringelbach, 2005; Wallis, 2007; Peters and Buchel, 2010; Rangel and Hare, 2010; Levy and Glimcher, 2012; Roy et al., 2012). Most notably, research suggests that vMPFC activity encodes the subjective values of various types of rewards (for review, see Peters and Buchel, 2010; Rangel and Hare, 2010; Levy and Glimcher, 2012; Sescousse et al., 2013). For example, neuroeconomic studies have shown that the vMPFC tracks the magnitude of monetary rewards and the idiosyncratic values subjects place on those rewards; activity in this area correlates with monetary reward outcome (Knutson et al., 2003), the subject-specific valuations of gains and losses (Tom et al., 2007), and subject-specific discounted reward value (Kable and Glimcher, 2007). vMPFC activity reflects the subjective value that an individual assigns to other types of stimuli as well, including primary rewards (e.g., food) and various types of goods and social rewards (O’Doherty et al., 2003; Chib et al., 2009; FitzGerald et al., 2009; Hare et al., 2009, 2010; Lin et al., 2012). Furthermore, it has been shown that damage to the vMPFC results in disturbances of subjective valuation (Moretti et al., 2009; Sellitto et al., 2010; Glascher et al., 2012). Together, these and related findings have led to the view that the vMPFC integrates information from multiple sources to represent the significance or value of stimuli (Wallis, 2007; Peters and Buchel, 2010; Rangel and Hare, 2010; Levy and Glimcher, 2012; Sescousse et al., 2013).

**Figure 1:**
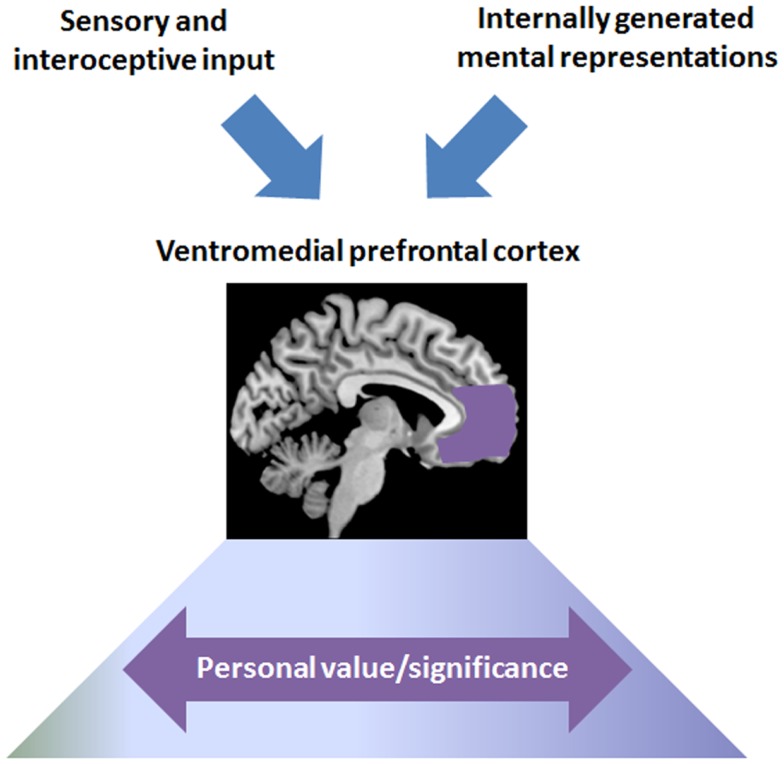
**Schematic representation of the function of the vMPFC according to the valuation hypothesis**. The vMPFC may represent the personal value or significance of various types of information, not only stimuli from the external and internal environment (i.e., sensory and interoceptive input) but also internally generated mental contents (e.g., thoughts, images, memories), including self-representations. Personal significance is processed along a continuum, such that stimuli and mental contents are assigned degrees of significance.

Although the medial prefrontal activations that have been related to value-based processing are sometimes confined to the most ventral part of the vMPFC (i.e., the medial orbitofrontal cortex), many neuroeconomic studies have reported activations that strikingly overlap with the vMPFC areas that are commonly detected in self-processing studies (see e.g., Kable and Glimcher, 2007; Chib et al., 2009; Hare et al., 2009). Neuroeconomic studies focused on the role of the vMPFC in the subjective valuation of stimuli from the external environment that are only loosely, if at all, related to self-representations. Yet the findings raise the possibility that vMPFC responses when processing self-related contents could reflect a similar valuation mechanism. Indeed, self-related contents are rarely considered in a dispassionate way: stimuli and mental representations that refer or relate to the self are assigned unique value and are associated with strong affective investments (James, 1890; Pelham, 1991; Leary, 2004). The function of the vMPFC during self-processing may precisely be to appraise or represent the personal value or significance^2^ of self-related contents, an idea that is here referred to as the “valuation hypothesis” (see Figure 1).

The idea that the vMPFC might signal the personal significance of self-related contents has been echoed by several researchers. Schmitz and Johnson (2007) have proposed that the vMPFC instantiates supramodal processes that contribute to detect the self-relevance of various types of stimuli. Northoff and Hayes (2011) discussed several ways in which self-relevance and value-based processing could be related, and argued that although the two processes may not be reducible to one other, they clearly interact and involve common neural substrates. The evidence reviewed by these authors, however, mainly focused on the self-relevance of external stimuli, such as pictures of emotional scenes or rewarding stimuli (e.g., Phan et al., 2004; de Greck et al., 2008; Enzi et al., 2009). Other researchers have proposed and provided more direct evidence that the vMPFC may also signal the personal significance of self-related mental contents, such as memories, prospective thoughts, and representations of one’s personality traits (Andrews-Hanna et al., 2010b; D’Argembeau et al., 2010a, 2012a). Finally, a recent meta-analysis has revealed the broad involvement of the vMPFC across studies of memory, self-representation, social cognition, emotion, reward, pain, and autonomic regulation (Roy et al., 2012). In an effort to unravel the common denominator to these seemingly disparate functions, Roy et al. argued that the vMPFC may integrate various sources of information to conceive the meaning of events for one’s well-being and future prospects.

A common theme across several proposals is therefore that the vMPFC encodes personal value or significance. This valuation mechanism may be applied to different kinds of information, not only stimuli from the external environment but also internally generated mental contents. From this perspective, the function of the vMPFC during self-processing may be to appraise or represent the significance of self-related information. To date, however, the extent to which activity changes in the vMPFC during self-processing tasks actually reflect the personal value that is assigned to self-related contents has not been examined in detail. In the next section, I discuss several lines of research that provide support for this hypothesis.

### Evidence for the valuation hypothesis

If the vMPFC contributes to assign personal value to self-related information, then the activity of this region should vary with the importance that an individual attaches to particular self-related contents at a given moment. Several lines of evidence suggest that this is indeed the case.

#### Taking distance from self-representations

One way to test the valuation hypothesis would be to experimentally manipulate the value that is assigned to self-representations and to examine whether the processing of these representations is associated with corresponding changes in vMPFC activity. Several studies have done this by investigating the effects of temporal distance on the neural correlates of self-processing. There is evidence that people value their current self to a greater extent than temporally distant selves (Wilson and Ross, 2001, 2003), such that they tend to treat their past and future selves as they would treat other individuals (Pronin and Ross, 2006; Pronin et al., 2008). If the vMPFC is involved in assigning value to self-representations, the activity of this region should be sensitive to these effects of temporal distance. In one fMRI study, we tested this hypothesis by comparing the neural correlates of making trait judgments about the present self versus a past self (D’Argembeau et al., 2008). Participants were instructed to reflect on their own traits and those of a close other, for both their present life period and a past life period (i.e., 5 years ago). We found that the degree of activity in the vMPFC varied significantly according to the target of reflection. Specifically, the vMPFC was more active when participants thought about their present self than when they thought about their past self or about the other person; thinking about the past self and thinking about the other person were associated with similar levels of activity. In a subsequent study (D’Argembeau et al., 2010b), we found that this effect of temporal distance was symmetrical between the past and the future: participants showed higher activity in the vMPFC when making trait judgments about their present self than when making trait judgments about themselves 5 years ago or 5 years from now (with no difference between past and future selves). These findings suggest that reducing the personal significance of self-representations (by increasing temporal distance) leads to corresponding decreases in vMPFC activity during self-referential thinking.

Other studies have shown that the magnitude of the differential activity in the vMPFC when thinking about present versus future selves correlates with individual differences in the propensity to devalue future rewards. During fMRI scanning, Ersner-Hershfield et al. (2009) asked participants to judge personality traits in reference to the self or another person for both the present and the future (i.e., 10 years from now). Approximately 1 week after the scanning session, participants returned to the laboratory to complete a temporal discounting task in which they had to make a series of binary choices between an immediate monetary gain and a delayed (but usually larger) gain. In line with the above-mentioned findings, a region of the vMPFC showed greater activation for present self trials than for future self and other trials. Furthermore, a measure of differences in neural activation in this region between present and future self trials correlated positively with individual estimates of temporal discounting. In other words, participants who displayed greater activity in the vMPFC when thinking about present versus future selves showed a greater propensity to devalue future rewards. Related findings have been reported in a study in which participants were scanned while they predicted how much they would enjoy engaging in each of a series of activities (e.g., spending the afternoon in a modern art museum) either in the present or in the future (a year later) (Mitchell et al., 2011). It was found that the vMPFC was more activated when predicting present compared to future enjoyment. Furthermore, differences in vMPFC activity between predictions of present and future enjoyment correlated positively with individual differences in the tendency to discount future monetary rewards, as assessed by intertemporal choice tasks. Thus, there is converging evidence that people who display greater reduction in vMPFC activity when thinking about future compared to present selves have a higher tendency to devalue future rewards. A plausible interpretation of this finding is that the vMPFC provides a signal reflecting the value that is placed on self-related contents for different time periods.

#### Psychological investment in self-representations

Perhaps the most direct evidence for the role of the vMPFC in representing the value of self-related information comes from a recent study that investigated the neural correlates of psychological investments in self-representations (D’Argembeau et al., 2012a). People have many different ideas and beliefs about who they are and what they are like, but they do not treat all self-views the same. Research has shown that we place more or less importance on particular self-views (our emotive investment) and hold different self-views with more or less confidence (our epistemic investment) (Pelham, 1991). For example, someone might attach much importance in being honest (high emotive investment), while considering that punctuality is not a particularly important trait for her to possess (low emotive investment); and for both traits, this person might feel more or less confident that she truly possesses these attributes (her epistemic investment). If the vMPFC represents the personal significance of self-related information, then the degree of neural activity in this region should correlate with one’s investments in self-representations, and in particular with emotive investments.

To test this hypothesis, we asked participants to make self-descriptiveness judgments regarding a variety of traits (e.g., honest, shy, punctual) while their brain activity was measured using fMRI (D’Argembeau et al., 2012a). Immediately after the scanning session, participants were presented again with the same set of traits and were instructed to rate the certainty of their self-representation regarding each trait (i.e., “how certain are you that you possess or do not possess this trait?”; from 1 = not at all, to 4 = completely), and the importance they attach to this self-representation (i.e., “how important is it for you to possess or not possess this trait?”; from 1 = not at all important, to 4 = very important). These ratings thus provided indexes of participants’ epistemic and emotive investments in each self-representation that had been processed during scanning. We then correlated the fMRI signal obtained during the self-descriptiveness judgments with the ratings of certainty and importance, which allowed us to identify the brain regions that responded to epistemic and emotive investments in self-representations on a trial-by-trial basis. The results showed that ratings of certainty and importance were correlated with neural activity in the MPFC, in both common and distinct MPFC areas. When looking at the brain regions that were specifically related to each kind of investment, we found that a region of the dMPFC responded specifically to the certainty of self-views, whereas a region of the vMPFC responded specifically to the importance of self-views. In other words, the level of activity of the vMPFC when participants contemplated their personal traits depended on their emotive investment in the particular self-representation under consideration: the higher the value attached to a self-representation, the stronger the response of the vMPFC. It should be reminded that participants did not explicitly reflect on the importance attached to their self-representations during scanning, such that the observed activity in the vMPFC is unlikely to reflect the engagement of explicit evaluation processes. Instead, the vMPFC might automatically confer degrees of value to the conceptions of the self that we form in our minds when we think about ourselves.

Other evidence suggests that the vMPFC is also involved in assigning personal significance to mental representations of events and facts from one’s life. In one fMRI study, we asked participants to imagine future events that were related to their personal goals (e.g., getting married next summer) and future events that were plausible and could be vividly imagined but were unrelated to their personal goals (e.g., taking a pottery lesson next summer), as determined by individualized pre-scan interviews (D’Argembeau et al., 2010a). We found that the vMPFC (as well as the PCC) showed greater activation when participants imaged future events that were related to their goals compared to future events that were unrelated to their goals. Importantly, these two types of future events were matched for vividness and temporal distance, suggesting that the observed differences in brain activation cannot be accounted by these factors. Instead, a plausible interpretation is that the increased activation of the vMPFC reflects the greater personal significance of events that are related to one’s goals. This interpretation receives some support from another study that analyzed the component processes subserved by different brain regions when people engaged in self-referential thinking. Andrews-Hanna et al. (2010b) found that the vMPFC and PCC were more activated when participants answered questions about various issues and events in their personal life (e.g., “Think about the major issues in your life at this moment. Which of these issues concerns you the most: health, education, or finance?”) than when they answered questions requiring the retrieval of general semantic knowledge (e.g., “At this moment there is a leading candidate in the Republican Party for President. Which of the following candidates is that candidate: Mitt Romney, Senator John McCain, or Rudy Giuliani?”). Additionally, various component processes that could be engaged when answering these different questions (e.g., mental imagery, recall of past experiences, affective processing, and so on) were assessed by an independent group of participants. This showed that three components were recruited to a greater extent when answering questions about the self: personal significance, evoked emotion, and introspection about one’s preferences, feelings, and emotions. When these three variables were combined into a composite score of “affective self-relevance,” this composite variable was found to account for a large portion of the trial-by-trial variance in activity within the vMPFC–PCC. The authors concluded that the vMPFC (along with the PCC) participates in evaluating aspects of personal significance.

#### Individual differences in valuing self-reflection

People differ in the extent to which they attach importance and manifest interest in introspecting about the self and their life (Trapnell and Campbell, 1999). In one study, we found that such individual differences modulate the activity of the vMPFC when people reflect on significant personal experiences (D’Argembeau et al., in press). Participants were asked to approach a set of personally significant memories in two different ways: on some trials, they remembered the concrete content of the events (e.g., what happened, where, when, with whom, and so on), whereas on other trials they reflected on the broader meaning and implications of their memories for the self (e.g., they thought about what the event says about their personality, how they have changed following this event, what they have learned, and so on). Individual differences in interest in this kind of self-reflection were assessed using a validated questionnaire that included items such as “I love exploring my inner self” (Trapnell and Campbell, 1999). We found that a number of brain regions (including the dMPFC, inferior frontal gyrus, middle temporal gyrus, and angular gyrus) were more activated when participants reflected on the meaning of their past experiences compared to when they remembered the concrete content of these experiences. The vMPFC was not consistently activated across participants but, interestingly, there was a positive correlation between the activity of the vMPFC and scores on the questionnaire assessing one’s interest in self-reflection. That is, the vMPFC showed increased activity when reflecting on the meaning of past experiences only for participants who have greater interest and willingness to introspect about the self.

#### Valuing outer aspects of self

As mentioned above, the vMPFC has been found to be more activated in response to objects that have been assigned to the self compared with objects that have been assigned to another person (Kim and Johnson, 2012, in press). Of particular interest, Kim and Johnson also found that the vMPFC was more activated in response to objects that were more preferred by the participants (as determined by post-scan ratings), but only for objects that had been assigned to the self. Furthermore, the participants’ willingness to trade their own objects for the other person’s objects was negatively correlated with vMPFC activity. These findings strongly suggest that the vMPFC represents the subjective value of self-related objects. Other studies have shown that the vMPFC responds to the self-relatedness of emotional or rewarding stimuli. Phan et al. (2004) found that the activation of the vMPFC in response to pictures of emotional scenes correlated with the extent to which participants associated to the pictures, especially (but not exclusively) when they explicitly reflected on the self-relatedness of the stimuli (see also Northoff et al., 2009). Related findings have been reported by de Greck et al. (2008) who found that the response of the vMPFC to pictures of rewarding stimuli (e.g., food items) was greater when the stimuli were judged to be high (compared to low) in self-relatedness (see also Enzi et al., 2009). As noted earlier, there is also evidence that the vMPFC is activated when thinking about persons that tend to be included in one’s sense of self, such as close others. From this finding, Krienen et al. (2010) concluded that the vMPFC contributes to “evaluate or provide a signal reflecting the personal significance of close others” (p. 13911). Taken together, these different studies suggest that the vMPFC represents the personal significance of a variety of outer self-related contents.

#### Summary

Although relatively few studies to date have directly tested the valuation hypothesis, several lines of evidence converge to suggest that vMPFC activity during self-processing depends on the personal significance of self-related contents. First, increasing psychological distance (in particular, temporal distance) from self-representations leads to decreased activity in the vMPFC during self-reflective thinking. Second, vMPFC activity increases linearly with the personal importance of the self-representations under consideration. Third, individual differences in the interest placed on self-reflection modulate the activity of the vMPFC during self-reflective thinking. Finally, the vMPFC responds to outer aspects of self that have high personal value, such as possessions and close others. Taken together, these findings provide support to the view that the vMPFC contributes to assign personal value to self-related information.

## Personal Significance and Psychological Health

Assigning personal value to self-related contents may be essential for constructing and stabilizing coherent self-representations (Markus, 1977; Pelham, 1991). Indeed, it has been suggested that disturbance in the brain’s systems that assign personal significance may contribute to the alterations of self boundaries that are observed in some psychiatric disorders (Feinberg, 2011). In addition, an excessive investment in, and identification with, rigid and dysfunctional self-views may also play an important role in depression and anxiety (Clark and Beck, 2010). It is therefore interesting to note that various forms of psychopathology are characterized by altered patterns of activity in the vMPFC during self-processing. For example, it has been found that the differential activity in the vMPFC when processing self-related compared to non-self-related contents is reduced in schizophrenia (Holt et al., 2011) and absent in autism (Lombardo et al., 2010); depression has been associated with both abnormal increases and decreases in vMPFC activity during self-processing (Lemogne et al., 2012); and patients with social anxiety disorder show atypical modulation of vMPFC activity in response to self-referential comments (Blair et al., 2011). An intriguing possibility is that these alterations in the functioning of the vMPFC may contribute to the abnormalities in the processing of personal significance that are observed in these different disorders.

Some interventions have proven their efficacy in addressing dysfunctional self-views and recent studies suggest that their effects may in part be mediated by a modulation of vMPFC activity during self-processing. Research has shown that the practice of mindfulness meditation – paying attention to one’s current experience in a non-evaluative way – has beneficial effects across diverse psychological disorders as well as for well-being (for review, see Brown et al., 2007; Keng et al., 2011). These salutary effects are likely due to multiple mechanisms of actions and may, in part, involve a change in perspective on the self (Holzel et al., 2011). By closely observing the contents of consciousness in a non-judgmental way, practitioners learn to see their thoughts and emotions as transient mental events. Through this process, one adopts a more detached perspective on the self, which may foster a disidentification from, and modification of, rigid and dysfunctional self-views (Holzel et al., 2011; Vago and Silbersweig, 2012) and may lead to more accurate self-knowledge (Carlson, 2013).

Farb et al. (2007) specifically investigated the neural correlates of such change in perspective on the self following mindfulness practice (for a comprehensive review of the neurobiological changes promoted by mindfulness, see Vago and Silbersweig, 2012). Participants who completed mindfulness training were compared with participants who had not yet undergone training while they engaged in two modes of self-reference: in one condition, they were asked to think about the personal meaning and self-descriptiveness of trait adjectives (referred to as “narrative” focus), whereas in another condition they were instructed to monitor their moment-to-moment experience in response to the adjectives (referred to as “experiential” focus). In line with the neuroimaging studies of self-processing reviewed above, narrative self-focus induced activation in several brain regions, including the vMPFC, in both groups of participants. Interestingly, however, individuals who completed the mindfulness training showed larger reductions in vMPFC activity during the experiential (compared with the narrative) focus, along with increased engagement of the right lateral prefrontal cortex, right insula, secondary somatosensory cortex, and inferior parietal lobule. The authors interpreted these findings as representing a shift “toward more lateral prefrontal regions supporting a more self-detached and objective analysis of interoceptive (insula) and exteroceptive (somatosensory cortex) sensory events, rather than their affective or subjective self-referential value” (Farb et al., 2007, p. 319). Another study has shown that mindfulness practice influences functional connectivity between the vMPFC and other regions involved in self-processing, which may in part “reflect a reduction in emotional appraisal during self-referent processes” (Taylor et al., 2013, p. 12). Although these results are compelling, it should be noted that a study in patients with social phobia failed to find significant changes in vMPFC activity during self-processing following mindfulness training (Goldin et al., 2012), so additional research (in both healthy individuals and in various psychological disorders) is needed to further examine the possible contribution of the vMPFC in mindfulness-induced changes in self-processing.

Other evidence suggests that modifications of patterns of vMPFC activity during self-processing may underlie the restructuration of dysfunctional self-views following cognitive-behavioral therapy in depression (Yoshimura et al., in press). A group of depressive patients underwent a cognitive-behavioral intervention program that involved, among other things, the identification and restructuration of negative self-views and the development of positive thinking about the self. The patients were scanned before and after the therapy while they made self-descriptiveness judgments on positive and negative traits. Before therapy, the patients showed higher activity in the vMPFC when considering negative compared with positive aspects of the self (see also Lemogne et al., 2012, for a review of studies showing abnormalities of MPFC activity during self-processing in depression). From pre- to post-therapy, there was a decrease in vMPFC activity when thinking about negative aspects of the self and an increase in vMPFC activity when thinking about positive aspects of the self, such that following therapy the patients recruited the vMPFC to a greater extent when considering positive compared with negative aspects of themselves. This shift in patterns of vMPFC activity from pre- to post-therapy may result from a restructuration of the relative value that the patients placed on their positive versus negative self-views, such that positive conceptions of the self are assigned greater significance following treatment.

## The vMPFC and Spontaneous Self-Related Thoughts

The vMPFC is a central hub of the default network – a set of interacting brain regions that show increased activity during “resting” states compared with active, externally focused tasks (Shulman et al., 1997; Gusnard and Raichle, 2001; Mazoyer et al., 2001; Buckner et al., 2008). Although the exact function of this network remains somewhat controversial, a prominent hypothesis is that it supports internal mentation during rest and passive task conditions (Buckner et al., 2008; Andrews-Hanna, 2012). When our attention is not focused on a given task, we spontaneously experience all sorts of thoughts and mental images: we may, for example, revisit a past event or think about things to do in the future (Smallwood et al., 2009; Andrews-Hanna et al., 2010a; Stawarczyk et al., 2011a). In line with the internal mentation hypothesis, a number of studies have linked this kind of spontaneous mental activity to the default network (McKiernan et al., 2006; Mason et al., 2007; Christoff et al., 2009; Andrews-Hanna et al., 2010a; Stawarczyk et al., 2011b).

Recent findings further suggest that the default network comprises multiple subsystems that likely support distinct component processes involved in internal mentation (Andrews-Hanna et al., 2010b). Of particular interest here is the finding that the resting state and explicit self-processing are associated with shared activation in the vMPFC. In a pioneering study, Andreasen et al. (1995) used positron emission tomography (PET) to investigate similarities and differences in neural activity between the explicit retrieval of autobiographical memories and a rest condition (i.e., lying quietly with no specific instructions about mental activity). They found that the vMPFC and precuneus showed greater activity during both autobiographical memory retrieval and rest compared to a semantic memory condition. Interviews with the participants indicated that they thought about a variety of things during the rest condition, but especially about self-related contents such as past experiences and future activities. The authors concluded that the psychological commonality between the rest and autobiographical memory conditions is that “both involve something personal and highly individual” (p. 1583).

Another PET study investigated the commonalities in brain activation between rest and the explicit reflection on one’s personality traits (D’Argembeau et al., 2005). It was found that both conditions were associated with common activation in the vMPFC compared with conditions requiring participants to reflect on non-self-related contents (see also Whitfield-Gabrieli et al., 2011). Furthermore, an analysis of the content of mental activity (using verbal reports and rating scales obtained after each scan) showed that participants spontaneously experienced self-referential thoughts during the rest condition and that the amount of self-referential processing correlated specifically with the activity of the vMPFC. A recent quantitative meta-analysis has confirmed that the resting state and explicit self-processing are associated with common activations in the vMPFC. Qin and Northoff (2011) compared the location of activations in studies on the default network (i.e., brain regions showing stronger activation during the resting state compared to active tasks) with the location of activations associated with various self-related tasks (e.g., trait judgments, autobiographical memory, face recognition, and name perception). These authors found that the resting state and self-related tasks showed overlapping activations in a region of the vMPFC.

Overall, these findings suggest that the vMPFC is engaged in both intentional (in explicit self-referential tasks) and spontaneous (in the resting state) self-processing. In light of the valuation hypothesis, an intriguing possibility is that the activation of the vMPFC during the resting state may signal the personal significance of spontaneous cognitions – be they memories, future thoughts, or other reflections on self-related contents. In this way, the vMPFC might contribute to highlight and select, among the many thoughts and mental images that spontaneously populate our minds in daily life, representations that are likely to have some relevance for guiding our decisions and behavior.

## Concluding Remarks and Future Directions

Despite extensive evidence that the vMPFC is involved in processing self-related contents, the precise function of this region is still elusive. Here I have suggested that a key dimension that may shed light on this issue is the notion of personal significance. Stimuli and mental representations that refer or relate to the self tend to be assigned unique value, and the function of the vMPFC may precisely be to evaluate or represent such significance. The notion of personal significance should be conceived as a continuum, such that some self-related contents are assigned more value than others. Furthermore, the personal value of a given content is probably not fixed once for all, but may instead vary according to the context and evolve across time. By flexibly assigning degrees of value to self-related contents, the vMPFC may play an important role in the construction, stabilization, and modification of self-representations, and ultimately in guiding our choices and decisions. Although this is certainly not the sole ingredient of our sense of self, the representation of personal significance in the vMPFC may contribute to establish the fundamental distinction between self and non-self that each of us subjectively experiences.

While the evidence reviewed in this article provides support to the valuation hypothesis, many questions remain and this hypothesis clearly requires further investigation. First, the notion of personal significance is admittedly quite vague and requires further refinement. The relevance of something can be considered at numerous levels – from basic physiological needs to higher-order goals, motives, and values – and it will be important in future work to dissect the precise dimensional features of relevance that may be represented in the vMPFC. A related question is whether personal significance can be entirely reduced to valuation, as defined in the neuroeconomic literature, or whether they represent (at least partly) dissociable processes (see Northoff and Hayes, 2011, for further discussion of this issue). Recent findings suggest that different dimensions of value (i.e., economic vs. core value) can be dissociated in the MPFC (Brosch et al., 2012) and that the vMPFC can process value independently of self-relevance (Nicolle et al., 2012). Thus, it remains to be determined whether the same valuation scale is applied to self-related versus non-self-related contents or whether the two types of contents are processed along qualitatively different dimensions of value. From this perspective, it will also be important to investigate whether and how different value dimensions are processed by the vMPFC and other brain structures that have been associated with relevance detection, such as the amygdala and ventral striatum (see e.g., Sander et al., 2003; Adolphs, 2010). Interestingly, it has been found that the vMPFC is functionally coupled to the amygdala and ventral striatum when processing self-related contents (Schmitz and Johnson, 2006), but the specific contribution of each of these areas remains to be determined.

Another question relates to the functional specialization within the vMPFC. In this article, I have considered the vMPFC as a whole but this is of course a fairly large area that comprises multiple subregions (Ongur et al., 2003). Activations in some neuroimaging studies of self-processing are quite extensive, encompassing multiple vMPFC subregions (e.g., D’Argembeau et al., 2010b), whereas other studies have reported activations in specific subregions, such as the rostral anterior cingulate cortex (e.g., Ersner-Hershfield et al., 2009), the rostral vMPFC (BA 10) (e.g., Benoit et al., 2010), or the medial orbitofrontal cortex (e.g., Hughes and Beer, 2012). It is likely that distinct areas within the vMPFC support different processes, some of which may not be directly related to the assignment of personal significance. However, the functional specialization within the vMPFC remains largely unexplored in the context of self-processing. Likewise, whether the exact same area(s) of vMPFC are involved in representing the value of self-related and non-self-related contents has not been directly investigated. Examining the functional properties of different vMPFC areas across species that differ in their ability to form self-representations, as well as at different developmental stages of self-representational abilities in humans, might shed some light on these questions.

This raises the broader question of whether a common valuation mechanism is applied to various kinds of stimuli and mental contents regardless of their peculiarities. Previous research suggests that the vMPFC encodes the subjective value of different types of rewards on a common neural scale (Peters and Buchel, 2010; Rangel and Hare, 2010; Levy and Glimcher, 2012), yet there also appears to be some functional specialization within the vMPFC according to types of rewards. In particular, the evidence suggests a posterior–anterior distinction within the vMPFC according to levels of abstraction, with more abstract rewards being represented more anteriorly than less abstract rewards (Kringelbach and Rolls, 2004; Bechara and Damasio, 2005; Sescousse et al., 2013). Self-representations can also be more or less abstract and it would be interesting to investigate whether these variations can be mapped onto a similar posterior–anterior axis within the vMPFC (see Martinelli et al., 2013, for recent evidence suggesting that this might in part be the case).

Finally, the role of automatic and controlled processes in the assignment of personal significance deserves further attention. The processing of personal significance is not necessarily conscious and deliberate, and in fact it likely operates outside of awareness most of the time (Bargh and Morsella, 2008). As already noted, current evidence suggests that the vMPFC may automatically confer degrees of value to self-related contents (D’Argembeau et al., 2012a), but of course this does not mean that this process cannot be modulated by conscious awareness; indeed, the research reviewed above suggests that mindfulness practice can lead to significant changes in how one approaches self-related contents. Identifying the exact conditions under which the processing of personal significance can be influenced by conscious monitoring processes, and the role of the vMPFC in this respect, is an important avenue for future research that could potentially deepen our understanding of healthy and unhealthy ways of relating to oneself.

## Conflict of Interest Statement

The authors declare that the research was conducted in the absence of any commercial or financial relationships that could be construed as a potential conflict of interest.

## Footnotes

^1^It should be noted that the designation of different portions of medial prefrontal cortex varies somewhat across studies. In this article, I use the term medial prefrontal cortex (MPFC) to refer to the entire central portion of the prefrontal cortex, including the anterior cingulate gyrus. The label ventromedial prefrontal cortex (vMPFC) is used to refer to a broad area in the lower central portion of the prefrontal cortex, encompassing medial sections of Brodmann’s areas (BA) 10, 11, and lower BA 32, whereas the label dorsomedial prefrontal cortex (dMPFC) refers to the higher portion of MPFC, encompassing medial sections of BAs 8, 9, and higher BA 32.

^2^By personal value or significance, I simply mean the worth or importance of something for a particular individual.
